# Dissemination of clinical *Escherichia coli* harboring the *mcr-1* gene in Pakistan

**DOI:** 10.3389/fmicb.2024.1502528

**Published:** 2025-01-07

**Authors:** Sabahat Abdullah, Muhammad Ahmad Mushtaq, Kalim Ullah, Brekhna Hassan, Mariya Azam, Muhammad Asif Zahoor, Juan Wang, Jianzhen Xu, Mark A. Toleman, Mashkoor Mohsin

**Affiliations:** ^1^Institute of Microbiology, University of Agriculture, Faisalabad, Pakistan; ^2^Department of Bioinformatics, Shantou University Medical College, Shantou, China; ^3^School of Medicine, Department of Medical Microbiology, Institute of Infection and Immunity, Cardiff University, Cardiff, United Kingdom; ^4^Institute of Molecular Biology and Biotechnology (IMBB), The University of Lahore, Lahore, Pakistan; ^5^Institute of Microbiology, Government College University, Faisalabad, Pakistan; ^6^Department of Preventive Veterinary Medicine, College of Veterinary Medicine, Northwest A&F University, Yangling, China

**Keywords:** *Escherichia coli*, Colistin, MCR-1, whole-genome sequencing, antibiotic resistance

## Abstract

**Background:**

Colistin is an antibiotic used as a last resort to treat multidrug-resistant Gram-negative bacterial infections. Plasmid-mediated mobile colistin-resistant (*mcr*) genes in *Escherichia coli* (*E. coli*) are disseminated globally and are considered to be a major public health threat. This study aimed to determine the molecular characteristics of colistin-resistant *Escherichia coli* isolates in clinical settings in Pakistan.

**Methods:**

A total of 240 clinical *E. coli* strains isolated from urine and pus cultures were collected from two hospitals in Faisalabad and analyzed for phenotypic resistance to colistin by cultivation on CHROMagar plates supplemented with colistin 2 ug/ml. Molecular characteristics of colistin-resistant isolates were analyzed using conventional PCR, whole genome sequencing, and bioinformatics analysis.

**Results:**

PCR and whole genome analysis confirmed the presence of the *mcr-1* gene in 10 *E. coli* isolates. The minimum inhibitory concentration for colistin ranged from 4 ug/ml to 32 ug/ml. ResFinder analysis revealed the presence of multiple resistance determinants conferring co-resistance to *β*-lactams, aminoglycosides, trimethoprim, sulfonamides, tetracycline, quinolones, florfenicol, and macrolides. Hybrid genomic assembly indicated that *mcr-1* is carried on IncI2 plasmids. Plasmid replicon typing indicated that IncI2-type plasmids (*n* = 10) were the most prevalent plasmids in these strains, followed by IncFIB (*n* = 8), IncFIC (*n* = 7), IncFIA (*n* = 6), IncFII (4), IncQ1 (*n* = 3), IncI1 (*n* = 1), IncY (*n* = 1), and IncN (*n* = 1). The Achtman MLST typing scheme revealed a large diversity of STs among the *mcr-1*-positive *E. coli*. VirulenceFinder analysis revealed the presence of numerous virulence factors ranging from 4 to 19.

**Conclusion:**

Our study revealed the emergence and dissemination of colistin-resistant *E. coli* isolates carrying *mcr-1* in hospital settings, posing a potential risk to anti-infective therapy. More efforts should be taken to monitor the prevalence of *mcr-1*-carrying bacteria in Pakistan.

## Introduction

Antimicrobial resistance (AMR) is a major global public health concern, which has made the effective treatment of an ever-increasing array of infectious diseases very challenging (Aljeldah, 2022). Among Gram-negative bacteria, *Escherichia coli* (*E. coli*) causes a wide range of clinical infections due to endemic gut carriage, multidrug resistance, and, most importantly, rapid acquisition/transfer of resistance traits by horizontal gene transfer (Sora et al., 2021). Globally, 4.95 million deaths were reported in 2019 due to multidrug-resistant infections, with 1.27 million directly attributed to AMR (Murray et al., 2022). Moreover, the emergence of extended-spectrum *β*-lactamase- and carbapenemase-producing *E. coli* has resulted in the reduced effectiveness of antimicrobial treatment (van Den Bunt et al., 2020), such that healthcare professionals are now considering alternative antimicrobials such as colistin.

Colistin (also known as polymyxin E) is a cationic polypeptide antibiotic that interacts with the outer membrane of the Gram-negative bacteria (El-Sayed Ahmed et al., 2020). Despite colistin once being avoided due to its nephro- and neurotoxicity, this drug has now become a last-resort antimicrobial agent for treating life-threatening infections caused by MDR Gram-negative bacteria (Chibabhai et al., 2023). A gradual increase in the prevalence of colistin resistance has been noted in the last few years. The situation gets exacerbated by the fact that colistin resistance mediated by the *mcr* genes gets rapidly disseminated across diverse bacteria by horizontal gene transfer (Shafiq et al., 2023). The predominant carriers of *mcr*-*1* were IncX4, IncI2, and IncHI2 plasmids, which are transferable and adaptive plasmid types with broad host ranges. These plasmids contributed to the spread of *mcr*-*1* across various sources and bacterial species (Anyanwu et al., 2023).

A comprehensive review revealed that the *mcr* gene has been identified in 47 countries/regions across 6 continents, with 95% of the cases attributed to the *mcr-1* variant in 2019 (Feng et al., 2023). In Pakistan, the *mcr-1* gene has been detected in *E. coli* isolated from wildlife, humans, poultry, chickens, dairy cows, and insects (Azam et al., 2020; Umair et al., 2023; Li et al., 2022). However, there remains a scarcity of data, especially from clinical settings in Pakistan. Although a number of studies exist, they primarily focus on phenotypic resistance patterns (Javed et al., 2020; Hameed et al., 2021; Bilal et al., 2020a), and data showing detailed genomic analyses are scarce (Li et al., 2021). The gap in literature underscores the need for comprehensive genomic studies to understand the prevalence, genetic diversity, and mechanisms of co-resistance in clinical *E. coli* isolates harboring *the mcr* gene. By addressing this gap, the aim of this study was to characterize a set of colistin-resistant *E. coli* clinical isolates recovered from hospital settings in Pakistan. The results presented here will contribute to updating the status of colistin resistance in the country and improve the monitoring and surveillance of MDR *E. coli* harboring *mcr-1* gene within hospitals.

## Materials and methods

### Isolation of colistin-resistant *Escherichia coli* strains

A total of 240 *E. coli* strains recovered from urine or pus culture were collected from laboratories of two tertiary care hospitals in Faisalabad in 2019 and 2020, as described previously (Abdullah et al., 2023). These isolates were subcultured on CHROMagar media plates supplemented with 2 μg/mL colistin and incubated overnight at 37°C to confirm the purity and isolation of colistin-resistant *E. coli* isolates.

Protein-based confirmation was carried out primarily to confirm the identity of bacterial species using a matrix-assisted laser desorption ionization-TOF (MALDI-TOF; Bruker Daltonics, Germany).

### Molecular characterization of the *mcr-1* gene

DNA of *E. coli* isolates was subjected to conventional PCR to screen the presence of the *mcr-1* gene using primers and conditions, as described previously (Uddin et al., 2022).

### Antimicrobial susceptibility testing

The minimum inhibitory concentration (MIC) of *E. coli* isolates positive for the *mcr-1* gene was determined using the broth microdilution. Antimicrobial susceptibility testing of all colistin-resistant *E. coli* isolates was performed by disk diffusion using the Mueller–Hinton agar plates against 12 antimicrobials. *E. coli* ATCC 25922 was used as a negative control strain. The interpretation of colistin susceptibility was based on the breakpoint value defined by EUCAST (v. 13.0; Satlin et al., 2020).

### Whole genome sequencing and bioinformatics analysis

Total gDNA was extracted from an overnight culture (2 mL) on a QIAcube automated system (Qiagen). Following extraction, gDNA was quantified using fluorometric methods with a Qubit (Thermo Fisher Scientific). The quality ratios of gDNA (A260/280 and 260/230) were determined using a NanoDrop Spectrophotometer (Thermo Fisher Scientific). Genomic DNA libraries were prepared for whole genome sequencing using the Nextera XT kit (Illumina), as described by the manufacturer. Paired-end sequencing was performed using the Illumina MiSeq platform (MiSeq Reagent V3 Kit; 2 × 300 cycles). For each *E. coli* isolate, at least 80× coverage was generated. Raw sequence reads were trimmed using Trim Galore, and the genomes were *de novo*-assembled into contigs using SPAdes (3.9.0) with a pre-defined kmers set. Raw reads were also assembled using the Geneious (10.0.9; Biomatters Ltd.) *de novo* assembler, set at medium sensitivity for analysis of paired Illumina reads. Geneious was used to map both sets of contigs to reference genes identified using the closest BLAST homology and was also used to annotate genes from the closest homologs in the NCBI Genome database. Resistance genes were identified using Resfinder within CGE (Florensa et al., 2022), and wgMLST profiles were generated using the CGE platform coupled with the PubMLST.org database (Uelze et al., 2020). Plasmids were identified within the genome assembly and typed using Plasmidfinder (Carattoli and Hasman, 2020).

To reconstruct full circular plasmid sequences, isolates were also sequenced by MinION sequencing (Oxford Nanopore Technologies Ltd., Oxford Science Park, United Kingdom). Large-scale bacterial gDNA was extracted. The DNA library was prepared by pooling all barcoded samples to aim for a final DNA concentration of >500 ng/μL, and 1 μL of RAD was added to the DNA. A final mixture of 75 μL (34 μL sequencing buffer, 30 μL water, and 11 μL DNA library) was loaded into the flow cell. The MinION device was connected to the MinKNOW GUI to obtain the reads. The raw data in fast5 format were base-called with the high-accuracy mode and demultiplexed using Guppy 4.2.2 (Ulrich, 2023). Unicycler (0.4.4) was used to yield hybrid assembly using both Illumina short reads and MinION long reads. This process included assembling the long reads with Flye v2.8 (Hu et al., 2024) and following five rounds of polishing using Pilon (Vu et al., 2022) with the Illumina short reads of the same sample. The comparisons of the complete *E. coli* plasmids were visualized using BRIG (Darphorn et al., 2021).

The single-nucleotide polymorphism (SNP)-based phylogenetic tree was generated by calling and filtering SNPs, site validation, and phylogeny based on a concatenated alignment of the high-quality SNPs. For inferring phylogeny, the analysis was run with the standard parameters, and the EC958 strain genome (GenBank accession number HG941718.1) was used as a reference sequence. The number of SNPs exhibited among closely related isolates was calculated using a distance matrix file generated as a result of phylogeny (Kaas et al., 2014). The output core alignment file was used to construct the maximum-likelihood tree with 1,000 bootstrap replications using MEGA-X v 10.0.5 (Kumar et al., 2018).^1^ The phylogenetic tree of the alignments was visualized and edited using iTOL v 4.4.2 software (Letunic and Bork, 2019).^2^

## Results

### Identification of *mcr*-positive *Escherichia coli*

Ten non-repetitive colistin-resistant *E. coli* strains were isolated from two hospitals over 1 year. These non-duplicated strains were mainly isolated from urine (70%) followed by pus (30%) cultures. Similarly, the percentage of colistin-resistant *E. coli* isolated from female and male samples was found to be 60 and 40%, respectively. The number of patients belonging to age ranges of 15–25 years, 35–45 years, 45–55 years, 55–65 years, and 65–75 years were 2, 3, 3, 1, and 1, respectively. All 10 colistin-resistant *E. coli* isolates were found to be *mcr-1* producers using specific *mcr-1* PCR.

### Antimicrobial resistance phenotypes

All of the isolates exhibited an MIC of colistin between 4 μg/mL and 32 μg/mL (Supplementary file 1). Antimicrobial susceptibility testing revealed that all 10 *E. coli* isolates were MDR strains. MDR was defined as acquired non-susceptibility to at least one agent in three or more antimicrobial categories (Magiorakos et al., 2012). Isolates exhibited resistance to fluoroquinolones (100%), tetracyclines (100%), trimethoprim (80%), various types of penicillins (40–100%), and cephalosporins (10–60%). Moreover, 80% of isolates were susceptible to amoxiclav and aminoglycosides, while 100% susceptibility was observed for carbapenem antibiotics (Supplementary file 2).

### Phylogenetic analysis

Whole genome sequencing provided comprehensive information for the isolates and their phylogenetic relationship. Phylogenetic relationships among *E. coli* isolates were determined, and a maximum likelihood phylogenetic tree based on concatenated alignments of high-quality SNPs from the core genome was constructed. The genome of the sequenced isolates covered 72% of the EC958 reference genome.

**Figure 1:**
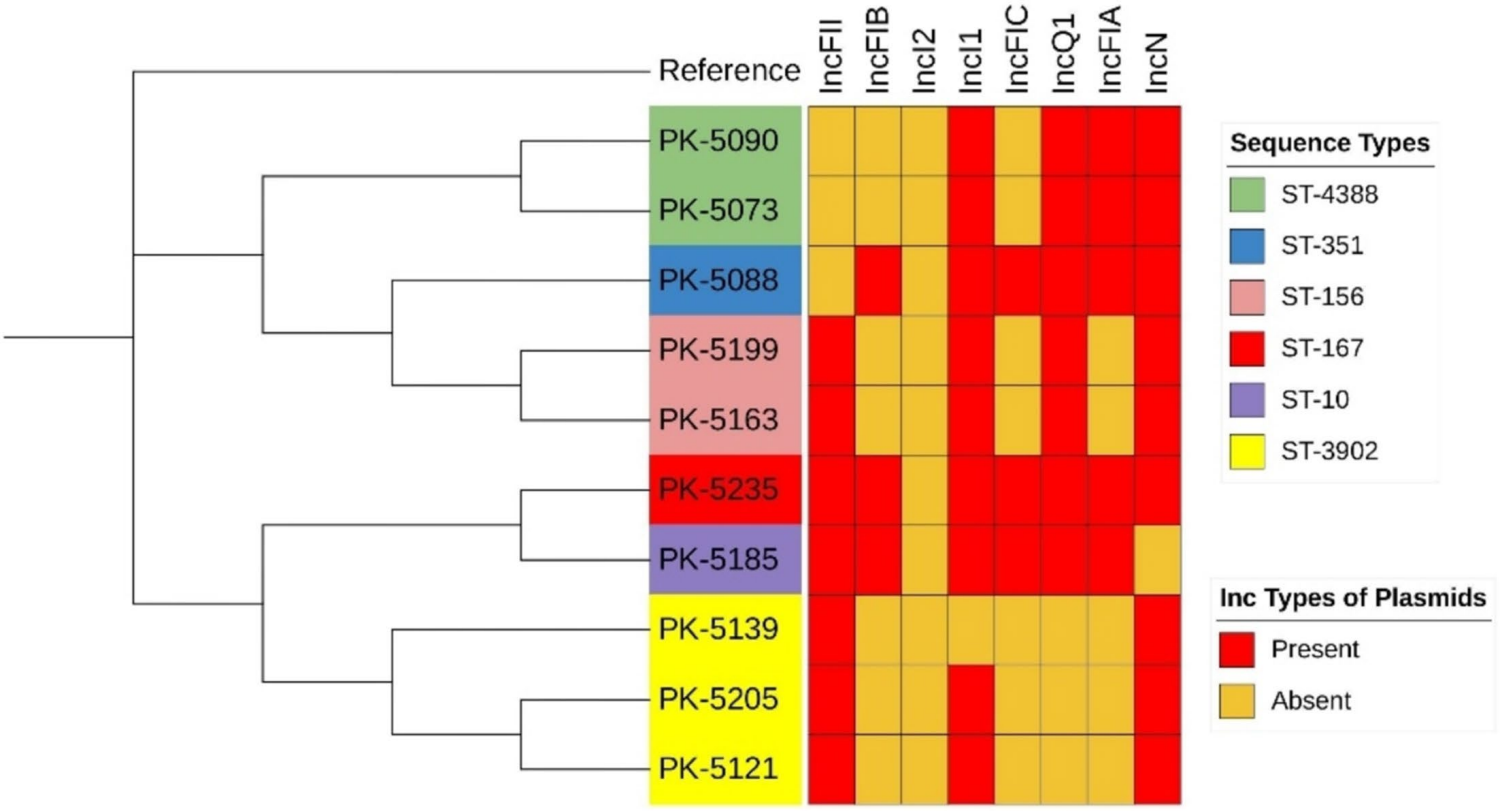
Phylogenetic tree of *mcr-1*-positive *Escherichia coli* isolates based on core genome SNP alignments.

We determined the SNP distance of the core genome. This method involves aligning the core genome sequences of the isolates, calling SNPs, and calculating SNP distances for comparing differences across *E. coli* isolates in the core genome, typically arising from whole-genome alignment and variant calling. The SNP matrix in the final dataset revealed a minimum of 27 SNPs and a maximum of 41,769 SNPs between all examined genomes. The core alignment showed that in ST-3902, isolates PK-5121 and PK-5139 differed from each other by 85 SNPs. These two isolates also differed by 277 and 256 SNPs, respectively, with the isolate PK-5235 from ST-167. Similarly, in ST-156, the isolates PK-5163 and PK-5199 differed by 45 SNPs (Figure 1; Supplementary file 3).

### *In silico* analysis of antimicrobial resistance genes and virulence genes

Whole genome analysis confirmed the presence of the *mcr-1* gene in 10 isolates. ResFinder analysis revealed the presence of multiple resistance genes for *β*-lactams, aminoglycosides, tetracycline, chloramphenicol, sulfonamides, trimethoprim, and quinolones, located on chromosomes and plasmids. Isolates carried antimicrobial resistance genes ranging from 5 to 14. PK-5185, containing 14 resistance genes, was the isolate with the largest number of resistance genes. The most prevalent resistance gene was floR, conferring resistance to florfenicol (*n* = 10). VirulenceFinder analysis showed the presence of various virulence factors (*n* = 22; *cia*, *cib*, *etsC*, *fyuA*, *gad*, *hlyF*, *hra*, *iroN*, *irp2*, *iss*, *iucC*, *iutA*, *lpfA*, *ompT*, *papA_F19*, *papC*, *sitA*, *terC*, *traT*, *cvaC*, *cma*, and *tsh*) in isolates ranging in number from 4 to 19 (Table 1).

**Table 1:** Genomic characteristics of clinical *Escherichia coli* isolates harboring the *mcr-1* gene.

Isolate ID	Inc type(s)	Resistance genes	Virulence genes
PK-5073	p0111_1, IncFIB(AP001918)_1, ColRNAI_1, ColE10_1, IncFII_1, IncI2_1_Delta, IncFIC(FII)_1, and Col(MG828)_1	*tet(X), bla_EC-18_, bla_TEM-215_, floR, fosA4, mph(A),* and *dfrA12*	*cvaC, etsC, gad, hlyF, iroN, iss, iucC, iutA, lpfA, mchF, ompT, sitA, terC, traT,* and *tsh*
PK-5088	p0111_1, ColE10_1, IncI2_1_Delta, ColRNAI_1, IncFII_1, and Col(MG828)_1	*tet(34), tet(A), bla_EC-18_, bla_TEM-215_, floR, catA1,* and *aph(3′)-Ia*	*gad, lpfA, terC,* and *traT*
PK-5090	IncFII_1, ColRNAI_1, IncFIB(AP001918)_1, ColE10_1, IncFIC(FII)_1, p0111_1, IncI2_1_Delta, and Col(MG828)_1	*tet(X), bla_EC-18_, bla_TEM-215_, floR, fosA4, mph(A),* and *dfrA12*	*cvaC, etsC, gad, hlyF, iroN, iss, iucC, iutA, lpfA, mchF, ompT, sitA, terC, traT,* and *tsh*
PK-5121	IncFIA_1, p0111_1, IncQ1_1, IncI2_1_Delta, IncFIB(AP001918)_1, ColRNAI_1, and IncFIC(FII)_1	*tet(34), tet(A), sul2, bla_EC-15_, bla_TEM-1_, floR, aph(3″)-Ib, aph(6)-Id, aph(3′)-Ia, dfrA14,* and *qnrS1*	*cia, cib, cma, cvaC, etsC, gad, hlyF, iroN, iss, iucC, iutA, ompT, papC, sitA, terC,* and *traT*
PK-5139	IncI1_1_Alpha, p0111_1, IncFIC(FII)_1, IncFIA_1, IncI2_1_Delta, IncQ1_1, IncFIB(AP001918)_1, and ColRNAI_1	*tet(34), tet(A), sul2, bla_EC-15_, bla_TEM-1_, floR, aph(3″)-Ib, aph(6)-Id, aph(3′)-Ia, dfrA14,* and *qnrS1*	*cia, cib, cma, cvaC, etsC, gad, hlyF, iroN, iss, iucC, iutA, ompT, papC, sitA, terC,* and *traT*
PK-5163	IncFIC(FII)_1, Col(MGD2)_1, ColRNAI_1, IncFIB(AP001918)_1, IncFIA_1, and IncI2_1	*tet(34), tet(A), sul2, bla_EC-18_, bla_TEM-1_, floR, aph(3″)-Ib, aph(6)-Id,* and *aph(3′)-Ia*	*cia, cib, etsC, fyuA, gad, hlyF, hra, iroN, irp2, iss, iucC, iutA, lpfA, ompT, papA_F19, papC, sitA, terC,* and *traT*
PK-5185	IncI2_1_Delta, Col(MGD2)_1, ColRNAI_1, and IncN_1	*tet(A), tet(34), tet(A), sul2, sul3, bla_EC_, bla_TEM-1_, floR, aph(3″)-Ib, aph(6)-Id, aadA2, aac(3)-Iid, mph(A),* and *mef(B)*	*gad, hra, papA_F19,* and *terC*
PK-5199	IncFIA_1, IncFIC(FII)_1, Col(MGD2)_1, IncI2_1_Delta, ColRNAI_1, and IncFIB(AP001918)_1	*tet(A), tet(34), sul2, bla_EC-18_, bla_TEM-1_, floR, aph(3″)-Ib, aph(6)-Id,* and *aph(3′)-Ia*	*cia, cib, etsC, fyuA, gad, hlyF, hra, iroN, irp2, iss, iucC, iutA, lpfA, ompT, papA_F19, papC, sitA, terC,* and *traT*
PK-5205	IncI2_1_Delta, IncFIC(FII)_1, IncFIA_1, IncFIB(AP001918)_1, p0111_1, IncQ1_1, and ColRNAI_1	*tet(34), tet(A), sul2, bla_EC-15_, bla_TEM-1_, floR, aph(6)-Id, aph(3″)-Ib, aph(3′)-Ia,* and *dfrA14*	*cia, cib, cma, cvaC, etsC, gad, hlyF, iroN, iss, iucC, iutA, ompT, papC, sitA, terC,* and *traT*
Pk-5235	Col(MG828)_1, ColRNAI_1, Col156_1, Col(BS512)_1, and IncI2_1_Delta,	*tet(34), sul2, bla_EC-15_, aph(6)-Id,* and *aph(3″)-Ib*	*capU, fyuA, gad, irp2, iss, terC,* and *traT*

### Plasmid characterization

Plasmid replicon typing exhibited 8 different Inc. types of plasmids, ranging from 1 to 10 per isolate. The incI2-type plasmid was observed to harbor the *mcr-1* gene among all *E. coli* isolates (*n* = 10; Table 2). The most prevalent Inc. types of plasmids observed were IncFIB, IncFIC, and IncFIA in 7, 7, and 5 numbers of isolates, respectively. IncFII and IncQ1 types were detected in three isolates each. Furthermore, IncI1 and IncN plasmid types were observed in a single isolate each.

**Table 2:** Characteristics of plasmids harboring the *mcr-1* gene.

Isolate ID	Plasmid type	Size (bp)	G + C content	Resistance gene
PK-5073	IncI2	63,227	48.5%	*mcr-1*
PK-5088	IncI2	64,819	45.6%	*mcr-1*
PK-5090	IncI2	56,636	43.1%	*mcr-1*
PK-5121	IncI2	54,509	48.4%	*mcr-1*
PK-5139	IncI2	58,676	42.5%	*mcr-1*
PK-5163	IncI2	15,589	46.7%	*mcr-1*
PK-5185	IncI2	59,013	42.8%	*mcr-1*
PK-5199	IncI2	59,609	49.9%	*mcr-1*
PK-5205	IncI2	57,157	46.8%	*mcr-1*
PK-5235	IncI2	29,093	47.7%	*mcr-1*

**Figure 2:**
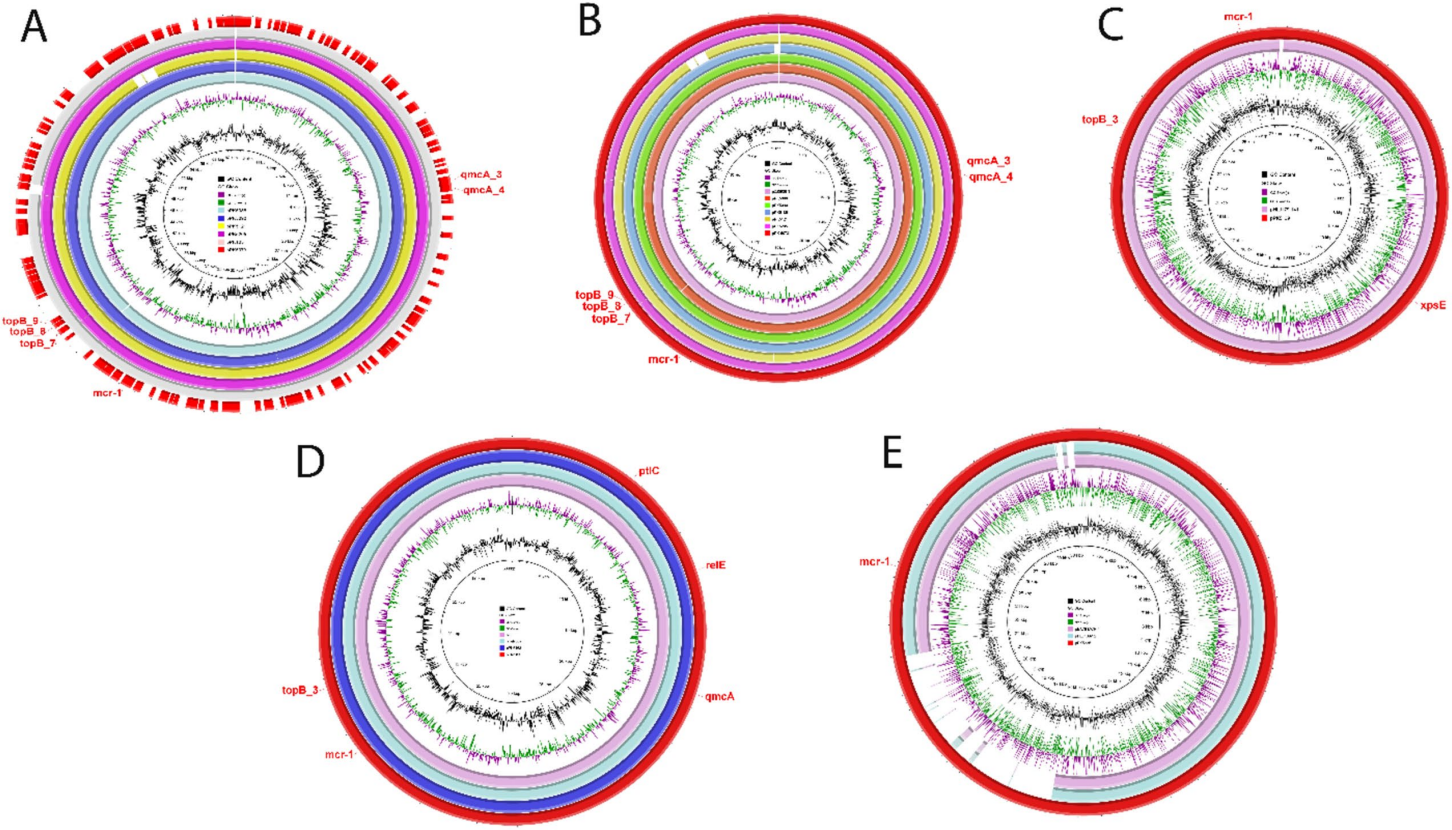
**(A–E)** Circular comparisons of IncI2 plasmid harboring the *mcr-1* gene with other different plasmids.

BLASTn analysis of plasmids harboring the *mcr-1* gene, pPK-5073, pPK-5088, pPK-5090, pPK-5121, and pPK-5205 against the NCBI nr database, showed that they exhibited homology with plasmid pPK105 (Accession no. MG808035.1), an *E. coli* strain isolated from chicken of healthy broiler in 2020 in Faisalabad, Pakistan, with identities of 94.98, 87.84, 94.57, 93.87, and 95.12 at a coverage of 97, 97, 98, 97, and 98%, respectively (Figure 2A). BLASTn analysis of these plasmids adding pPK-5185 against the NCBI nr database also exhibited homology with plasmid pZJ3920-3 (Accession no. CP020548.1) of *E. coli* strain isolated from the human bile in 2015 in Hangzhou, China, with identities of 95.32, 90.53, 94.98, 93.74, 93.63, and 94.98 at a coverage of 98, 98, 99, 98, 99, and 99%, respectively (Figure 2B). *E. coli* plasmid pHLJ179-141 (Accession no. MN232210.1) isolated from the chicken gut in 2019 in Guangzhou, China, had a backbone structure similar to plasmid pPK-5139 with identities of 94.76% and at a coverage of 99% (Figure 2C). BLAST search of pPK-5163 and pPK-5199 exhibited identity 91.97 and 100% at coverage of 99 and 96.85% with plasmid pHeN867 (Accession no. KU934208.1) of *E. coli* strain HeN867 isolated from chicken samples in China in 2020 and plasmid pC2 (Accession no. CP042471.2) of *E. coli* strain A50 isolated from *Gallus gallus* domesticus ISA15 in Algeria in 2017 (Figure 2D). Plasmid pPK-5235 shared a coverage of 80% and an identity of >95% with plasmid pHLJ109-92 (Accession no. MN232203.1) in *E. coli* strain isolated from the chicken gut in China in 2020 and plasmid pBA76-MCR-1 (Accession no. KX013540.1) in *E. coli* strain BA76 isolated from sacral wound swab in Bahrain in 2015 (Figure 2E).

## Discussion

Owing to the increasing incidence of carbapenemase-producing Enterobacteriaceae (Abdullah et al., 2023), colistin has become a last-resort antimicrobial, largely used for the treatment of severe bacterial infections. An additional challenge when it comes to polymyxin resistance is the rapid dissemination of MCR-producing Enterobacteriaceae. The emergence of *mcr-1*-producing *E. coli* isolates in hospital settings is a significant public health concern (Xie et al., 2022). Various approaches exist for detecting colistin resistance and *mcr* genes, each with unique strengths and limitations. Culture-based methods, such as broth microdilution, remain the gold standard for colistin susceptibility but are labor-intensive and costly. Rapid tests such as the colistin drop and colistin agar spot tests offer quicker alternatives but may suffer from accuracy issues, particularly with false-negative results. PCR-based and other nucleic acid amplification tests (NAATs) enable the rapid detection of the *mcr* genes directly from samples but rely on known gene variants, potentially missing novel or rare mutations. Whole genome sequencing (WGS) provides comprehensive resistance profiling, but its high cost and bioinformatics requirements may limit its use in routine diagnostics, especially in low-resource settings. The limited detection capabilities of many assays underscore the need for methodological advancements to improve the accuracy and accessibility of *mcr*-mediated colistin resistance screening (Zhang et al., 2022).

In this study, whole genome sequencing (WGS) was used to characterize clinical *E. coli* isolates harboring the *mcr-1* gene, a plasmid-mediated colistin resistance determinant, collected from two tertiary care hospitals in Pakistan. This study revealed the presence of the *mcr-1* gene in colistin-resistant *E. coli* isolates (*n* = 10/240, 4%). Other studies reported 2.6 and 2.8% prevalence of *mcr* genes in *E. coli* isolates in clinical settings in Pakistan (Bilal et al., 2020b; Ejaz et al., 2021). The findings of other studies performed in India and Iran also correspond to the results of our study and reported a 3.2 and 3% prevalence of *mcr* genes among clinical *E. coli* isolates (Wise et al., 2018; Aghapour et al., 2019). Several studies, particularly in Asia, documented a significant decrease in the emergence rate of isolates resistant to colistin due to the ban on its use to avoid the further emergence of isolates harboring the plasmid-mediated *mcr-1* gene (Binsker et al., 2022; Saeed et al., 2021; Wang et al., 2020; Usui et al., 2021). The relatively low proportion of *mcr-1*-producing *E. coli* isolates in hospital settings in this and other studies may reflect the limited use of colistin in clinical practice as opposed to the higher use of colistin in agriculture with corresponding higher proportions of resistant isolates among food-producing animals and agricultural communities (Lencina et al., 2024; Li et al., 2023). Countries including the United States, the United Kingdom, and Canada also never approved the use of colistin in livestock farming and have constantly described lower rates of detection of colistin-resistant isolates (Anwar et al., 2022; Binsker et al., 2022). Nevertheless, no such regulation on the ban of veterinary use of colistin was implemented in Pakistan, where colistin is frequently used in different antimicrobial combinations in animal farming for prophylactic and therapeutic purposes. Despite the low prevalence, continuous surveillance and strict control measures are vital to prevent the rapid spread of *mcr*-mediated resistance through horizontal gene transfer, preserving colistin’s role as a last-resort antibiotic.

It is noteworthy that no isolates in this study showed colistin resistance due to chromosomal mutations, which often contribute more frequently to resistance than the plasmid-mediated *mcr* genes. Typically, colistin resistance can arise from mutations in chromosomal genes such as *pmrA*, *pmrB*, *phoP*, *phoQ*, or *mgrB*, which alter the bacterial outer membrane by modifying lipopolysaccharides. These mutations are often observed in clinical isolates and can confer resistance without the need for *mcr* genes (Tkadlec et al., 2021). This is in agreement with the majority of the studies in Pakistan (Umair et al., 2023; Li et al., 2022).

Surveillance of AMR rates is a valuable tool to aid physicians in understanding the local resistance trends and improving the prescribing of antimicrobials. This study showed the resistance pattern in *E. coli* strains carrying the *mcr-1* gene isolated from pus (30%) or urine (70%) samples in hospital settings. The strains exhibited resistance to fluoroquinolones (100%), tetracyclines (100%), trimethoprim (80%), various types of penicillins (40–100%), and cephalosporins (10–60%). These findings are in agreement with the recent studies reporting increasing AMR in the region (Chaurasia et al., 2019; Browne et al., 2021). A recent study analyzed the AMR rates for GLASS-specified pathogen/antimicrobial combinations from Pakistan (2006–2018) and reported high resistance rates (>50%) to fluoroquinolones and third-generation cephalosporins among the *E. coli* and *K. pneumoniae* (Saeed et al., 2021). The emergence and spread of resistant pathogens are driven by factors such as high antibiotic selection pressure and poor infection control, often resulting in cross-transmission among patients (Castro-Sánchez et al., 2016). In this study, several *mcr-1* isolates also carried genes conferring resistance to *β*-lactams, aminoglycosides, and fluoroquinolones, complicating treatment options and underscoring the adaptive potential of *E. coli* under antibiotic pressure. Although data here are limited, these findings highlight the importance of antimicrobial stewardship and the need to explore alternative treatments.

The AMR gene profiles largely correlated with the phenotypic AST results, demonstrating resistance in line with the detected genes. For example, *mcr-1* correlated with colistin resistance, and the presence of β-lactamase genes (e.g., *bla_TEM-1_* and *bla_EC-18_*) matched with β-lactam resistance in AST. Aminoglycoside and tetracycline resistance genes (e.g., *aph(6)-Id* and *tet(A)*) similarly corresponded to observed phenotypic resistance. However, a few isolates showed susceptibility to certain antibiotics (e.g., aminoglycosides and amoxiclav) despite carrying resistance genes, suggesting potential gene expression variability or regulatory influences affecting the phenotype.

Understanding the occurrence of specific virulence genes among colistin-resistant *E. coli* isolates in this study sheds light on their potential pathogenicity. Predominant genes identified, such as *terC*, *gad*, *traT*, and *iss*, are linked to immune evasion and stress resistance, while *hlyF*, *iucC*, and *iroN* enhance iron acquisition, critical for survival in host environments. The co-presence of these virulence traits with colistin resistance highlights the heightened risk posed by these isolates, suggesting a need for more focused monitoring and tailored infection control strategies in healthcare settings.

The present study also evaluated the prevalence of STs in colistin-resistant *E. coli* clinical isolates. The MLST typing analysis of colistin-resistant *E. coli* isolates reveals a significant genetic diversity, with the isolates distributed across six different sequence types (STs). While the presence of multiple isolates from the sequence type ST-3902 might hint at its resilience under selective pressures, such as colistin use. In addition, colistin use alone may not be sufficient to drive the expansion of this particular ST over others, as clonal spread can result from various ecological and clinical factors beyond antibiotic selection. The presence of isolates within ST-4388 and ST-156 further indicates the prevalence of these lineages, while the unique STs (ST-351, ST-10, and ST-167) highlight the genetic variability among colistin-resistant *E. coli* isolates. The most common ST among clinical isolates of colistin-resistant *E. coli* was ST10, with a frequency of 11.63% worldwide (Dadashi et al., 2022). A notable aspect of the study involves examining the links between the colistin-resistant *E. coli* strains found in environmental and agricultural settings, particularly poultry, and those identified in clinical settings (Zahra et al., 2018; Tang et al., 2019). It is crucial to highlight that specific sequence types such as ST10 and ST156 have been observed in both contexts. This overlap suggests a potential zoonotic transmission pathway, indicating that the use of colistin in agriculture may contribute to the emergence and spread of resistant strains in humans. The presence of shared STs between environmental, agricultural, and clinical settings highlights the urgent need for coordinated AMR surveillance and control strategies across both sectors.

Numerous studies have reported various Inc-type plasmids carrying *mcr-1*; however, IncI2, IncHI2, and IncX4 are the three major types found in different species of Enterobacteriaceae (Aworh et al., 2021; Binsker et al., 2023; Vu et al., 2022). Notably, this study found the IncI2 plasmid type harboring the *mcr-1* gene among all isolates (*n* = 10), suggesting a strong association between IncI2 plasmids and the *mcr-1* gene, which confers colistin resistance. The most prevalent plasmid types included IncFIB, IncFIC, and IncFIA, indicating that these plasmid types are common carriers of resistance genes. In addition, IncFII, IncQ1, IncI1, and IncN types were also found among isolates. This diversity of plasmid types, especially the prevalence of various IncF plasmids, underscores the complex mechanisms of horizontal gene transfer contributing to the spread of colistin resistance.

## Conclusion and recommendations

The whole genome-based characterization of *mcr-1*-producing clinical *E. coli* isolates in Pakistan provides critical insights into the genetic diversity, resistance mechanisms, and transmission dynamics of these pathogens. By identifying similarities in resistance genes and virulence factors, the findings provide indirect insights into how these pathogens may spread across different settings. Colistin, often considered a last-resort antibiotic for MDR infections, is rendered ineffective by the presence of the *mcr-1* gene, leaving limited therapeutic options. The spread of such resistant pathogens could lead to increased morbidity, mortality, and healthcare costs. Based on our findings, we recommend implementing enhanced surveillance programs to monitor the prevalence and spread of *mcr-1*-producing *E. coli* and other resistant pathogens. Additionally, promoting the rational use of antibiotics through antimicrobial stewardship programs is essential to reduce selective pressure and prevent the emergence of resistance. We also advocate for increased investment in research to develop new antibiotics and alternative therapies, as well as rapid diagnostic tools for the timely detection of resistance genes.

## Data Availability

The genome sequences generated in this study are available in the NCBI GenBank database under accession numbers JBGKBF000000000, JBGKBG000000000, JBGKBH000000000, JBGKBI000000000, JBGKBJ000000000, JBGKBK000000000, JBGKBL000000000, JBHEFN000000000, JBHEFO000000000, and JBHEFP000000000.

## Footnotes

^1^ https://www.megasoftware.net/home

^2^ https://itol.embl.de

## References

[ref1] Abdullah S. Almusallam A. Li M. Mahmood M. S. Mushtaq M. A. Eltai N. O.. (2023). Whole genome-based genetic insights of Bla NDM producing clinical *E. coli* isolates in hospital settings of Pakistant. Microbiol Spectrum 11, e00584–e00523. doi: 10.1128/spectrum.00113-24 PMC10581159 37668386

[ref2] Aghapour Z. Gholizadeh P. Ganbarov K. Bialvaei A. Z. Mahmood S. S. Tanomand A.. (2019). Molecular mechanisms related to colistin resistance in Enterobacteriaceae. Infect. Drug Resist. 12, 965–975. doi: 10.2147/IDR.S199844, PMID: 31190901 PMC6519339

[ref3] Aljeldah M. M. (2022). Antimicrobial resistance and its spread is a global threat. Antibiotics 11:1082. doi: 10.3390/antibiotics11081082, PMID: 36009948 PMC9405321

[ref4] Anwar M. A. Aziz S. Ashfaq K. Aqib A. I. Shoaib M. (2022). Trends in frequency, potential risks and antibiogram of *E. coli* isolated from semi-intensive dairy systems. Pak. Vet. J. 42, 167–172. doi: 10.29261/pakvetj/2022.018

[ref5] Anyanwu M. U. Jaja I. F. Okpala C. O. R. Njoga E. O. Okafor N. A. Oguttu J. W. (2023). Mobile colistin resistance (mcr) gene-containing organisms in poultry sector in low-and middle-income countries: epidemiology, characteristics, and one health control strategies. Antibiotics 12:1117. doi: 10.3390/antibiotics12071117, PMID: 37508213 PMC10376608

[ref6] Aworh M. K. Kwaga J. K. Hendriksen R. S. Okolocha E. C. Thakur S. (2021). Genetic relatedness of multidrug resistant *Escherichia coli* isolated from humans, chickens and poultry environments. Antimicrob. Resist. Infect. Control 10, 1–13. doi: 10.1186/s13756-021-00930-x, PMID: 33757589 PMC7988975

[ref7] Azam M. Mohsin M. Johnson T. J. Smith E. A. Johnson A. Umair M.. (2020). Genomic landscape of multi-drug resistant avian pathogenic *Escherichia coli* recovered from broilers. Vet. Microbiol. 247:108766. doi: 10.1016/j.vetmic.2020.108766, PMID: 32768218

[ref8] Bilal H. Hameed F. Khan M. A. Khan S. Yang X. Rehman T. U. (2020a). Detection of mcr-1 gene in extended-spectrum β-lactamase-producing *Klebsiella pneumoniae* from human urine samples in Pakistan. Jundishapur J. Microbiol 13:646. doi: 10.5812/jjm.96646

[ref9] Bilal H. Tu R. Khan M. Hameed F. Zg J. (2020b). Discovery of mcr-1 harboring Incl2 plasmids from clinical isolates of multiclonal *E. coli* prevalent in Pakistan. Research Square.

[ref10] Binsker U. Käsbohrer A. Hammerl J. A. (2022). Global colistin use: a review of the emergence of resistant Enterobacterales and the impact on their genetic basis. FEMS Microbiol. Rev. 46:46. doi: 10.1093/femsre/fuab049 PMC8829026 34612488

[ref11] Binsker U. Oelgeschläger K. Neumann B. Werner G. Käsbohrer A. Hammerl J. A. (2023). Genomic evidence of mcr-1.26 IncX4 plasmid transmission between poultry and humans. Microbiol Spectrum 11, e01015–e01023. doi: 10.1128/spectrum.01015-23 PMC10434184 37358464

[ref12] Browne A. J. Chipeta M. G. Haines-Woodhouse G. Kumaran E. P. A. Hamadani B. H. K. Zaraa S.. (2021). Global antibiotic consumption and usage in humans, 2000–18: a spatial modelling study. Lancet. Planet. Heal. 5:e893. doi: 10.1016/S2542-5196(21)00280-1, PMID: 34774223 PMC8654683

[ref13] Carattoli A. Hasman H. (2020). PlasmidFinder and in silico pMLST: identification and typing of plasmid replicons in whole-genome sequencing (WGS). Horizontal gene transfer: methods and protocols. 2075, 285–294. doi: 10.1007/978-1-4939-9877-7_20, PMID: 31584170

[ref14] Castro-Sánchez E. Moore L. S. P. Husson F. Holmes A. H. (2016). What are the factors driving antimicrobial resistance? Perspectives from a public event in London. England. BMC Infect. Dis. 16, 1–5. doi: 10.1186/s12879-016-1810-x, PMID: 27590053 PMC5010725

[ref15] Chaurasia S. Sivanandan S. Agarwal R. Ellis S. Sharland M. Sankar M. J. (2019). Neonatal sepsis in South Asia: huge burden and spiralling antimicrobial resistance. BMJ 364:k5314. doi: 10.1136/bmj.k5314, PMID: 30670451 PMC6340339

[ref16] Chibabhai V. Bekker A. Black M. Demopoulos D. Dramowski A. du Plessis N. M.. (2023). Appropriate use of colistin in neonates, infants and children: interim guidance. South. Afr. J. Infect. Dis. 38:555. doi: 10.4102/sajid.v38i1.555, PMID: 38223435 PMC10784269

[ref17] Dadashi M. Sameni F. Bostanshirin N. Yaslianifard S. Khosravi-Dehaghi N. Nasiri M. J.. (2022). Global prevalence and molecular epidemiology of mcr-mediated colistin resistance in *Escherichia coli* clinical isolates: a systematic review. J. global antimicrobial resistance 29, 444–461. doi: 10.1016/j.jgar.2021.10.022, PMID: 34788692

[ref18] Darphorn T. S. Bel K. Koenders-van B. Brul S. ter B. (2021). Antibiotic resistance plasmid composition and architecture in *Escherichia coli* isolates from meat. Sci. Rep. 11:2136. doi: 10.1038/s41598-021-81683-w, PMID: 33483623 PMC7822866

[ref19] Ejaz H. Younas S. Qamar M. U. Junaid K. Abdalla A. E. Abosalif K. O. A.. (2021). Molecular epidemiology of extensively drug-resistant mcr encoded Colistin-resistant bacterial strains co-expressing multifarious β-lactamases. Antibiot 10:467. doi: 10.3390/antibiotics10040467 PMC8073099 33923991

[ref20] El-Sayed Ahmed M. A. E. G. Zhong L. L. Shen C. Yang Y. Doi Y. Tian G. B. (2020). Colistin and its role in the era of antibiotic resistance: an extended review (2000–2019). Emerg. microbes & infections 9, 868–885. doi: 10.1080/22221751.2020.1754133, PMID: 32284036 PMC7241451

[ref21] Feng J. Xu Z. Zhuang Y. Liu M. Luo J. Wu Y.. (2023). The prevalence, diagnosis, and dissemination of mcr-1 in colistin resistance: progress and challenge. Decoding Infection and Transmission 1:100007. doi: 10.1016/j.dcit.2023.100007

[ref22] Florensa A. F. Kaas R. S. Clausen P. T. L. C. Aytan-Aktug D. Aarestrup F. M. (2022). ResFinder–an open online resource for identification of antimicrobial resistance genes in next-generation sequencing data and prediction of phenotypes from genotypes. Microbial genomics 8:000748. doi: 10.1099/mgen.0.000748, PMID: 35072601 PMC8914360

[ref23] Hameed F. Khan M. A. Bilal H. Muhammad H. Rehman T. U. (2021). Detection of MCR-1 gene in multiple drug resistant escherichia coli and *klebsiella pneumoniae* in human clinical samples from Peshawar, Pakistan. Comb. Chem. High Throughput Screen. 24, 737–742. doi: 10.2174/1386207323666200914100119, PMID: 32928079

[ref24] Hu J. Wang Z. Sun Z. Hu B. Ayoola A. O. Liang F.. (2024). NextDenovo: an efficient error correction and accurate assembly tool for noisy long reads. Genome. Biol. 25:107. doi: 10.1186/s13059-024-03252-4 38671502 PMC11046930

[ref25] Javed H. Saleem S. Zafar A. Ghafoor A. Shahzad A. B. Ejaz H.. (2020). Emergence of plasmid-mediated mcr genes from gram-negative bacteria at the human-animal interface. Gut Pathogens 12:54. doi: 10.1186/s13099-020-00392-3, PMID: 33292525 PMC7678191

[ref26] Kaas R. S. Leekitcharoenphon P. Aarestrup F. M. Lund O. Friedrich A. (2014). Solving the problem of comparing whole bacterial genomes across differentsequencing platforms. PLoS One 9:e104984. doi: 10.1371/journal.pone.0104984, PMID: 25110940 PMC4128722

[ref27] Kumar S. Stecher G. Li M. Knyaz C. Tamura K. (2018). MEGA X: molecular evolutionary genetics analysis across computing platforms. Mol. Biol. Evol. 35, 1547–1549. doi: 10.1093/molbev/msy096, PMID: 29722887 PMC5967553

[ref28] Lencina F. A. Bertona M. Stegmayer M. A. Olivero C. R. Frizzo L. S. Zimmermann J. A.. (2024). Prevalence of colistin-resistant *Escherichia coli* in foods and food-producing animals through the food chain: a worldwide systematic review and meta-analysis. Heliyon. 10:e26579. doi: 10.1016/j.heliyon.2024.e26579, PMID: 38434325 PMC10904249

[ref29] Letunic I. Bork P. (2019). Interactive tree of life (iTOL) v4: recent updates and new developments. Nucleic Acids Res. 47, W256–W259. doi: 10.1093/nar/gkz239, PMID: 30931475 PMC6602468

[ref30] Li R. Lu X. Munir A. Abdullah S. Liu Y. Xiao X.. (2022). Widespread prevalence and molecular epidemiology of tet (X4) and mcr-1 harboring *Escherichia coli* isolated from chickens in Pakistan. Sci. Total Environ. 806:150689. doi: 10.1016/j.scitotenv.2021.150689, PMID: 34599956

[ref31] Li R. Mohsin M. Lu X. Abdullah S. Munir A. Wang Z. (2021). Emergence of plasmid-mediated resistance genes tet (X) and mcr-1 in *Escherichia coli* clinical isolates from Pakistan. Msphere 6, 10–1128. doi: 10.1128/mSphere.00695-21, PMID: 34431695 PMC8386413

[ref32] Li X. Zhu X. Xue Y. (2023). Drug resistance and genetic relatedness of *Escherichia coli* from mink in Northeast China. Pak. Vet. J. 43, 824–827. doi: 10.29261/pakvetj/2023.062

[ref33] Magiorakos A. P. Srinivasan A. Carey R. B. Carmeli Y. Falagas M. E. Giske C. G.. (2012). Multidrug-resistant, extensively drug-resistant and pandrug-resistant bacteria: an international expert proposal for interim standard definitions for acquired resistance. Clin. Microbiol. Infect. 18, 268–281. doi: 10.1111/j.1469-0691.2011.03570.x, PMID: 21793988

[ref34] Murray C. J. Ikuta K. S. Sharara F. Swetschinski L. Robles G. Gray A.. (2022). Global burden of bacterial antimicrobial resistance in 2019: a systematic analysis. Lancet 399, 629–655. doi: 10.1016/S0140-6736(21)02724-0, PMID: 35065702 PMC8841637

[ref35] Saeed D. K. Farooqi J. Shakoor S. Hasan R. (2021). Antimicrobial resistance among GLASS priority pathogens from Pakistan: 2006–2018. BMC Infect. Dis. 21, 1–16. doi: 10.1186/s12879-021-06795-0, PMID: 34876041 PMC8650393

[ref36] Satlin M. J. Lewis J. S. Weinstein M. P. Patel J. Humphries R. M. Kahlmeter G.. (2020). Clinical and laboratory standards institute and European committee on antimicrobial susceptibility testing position statements on polymyxin B and colistin clinical breakpoints. Clin. Infect. Dis. 71, e523–e529. doi: 10.1093/cid/ciaa121, PMID: 32052041

[ref37] Shafiq M. Bilal H. Permana B. Xu D. Cai G. Li X.. (2023). Characterization of antibiotic resistance genes and mobile elements in extended-spectrum β-lactamase-producing *Escherichia coli* strains isolated from hospitalized patients in Guangdong, China. J. Appl. Microbiol. 134:lxad125. doi: 10.1093/jambio/lxad125, PMID: 37336594

[ref38] Sora V. M. Meroni G. Martino P. A. Soggiu A. Bonizzi L. Zecconi A. (2021). Extraintestinal pathogenic *Escherichia coli*: virulence factors and antibiotic resistance. Pathogens 10:1355. doi: 10.3390/pathogens10111355, PMID: 34832511 PMC8618662

[ref39] Tang B. Chang J. Cao L. Luo Q. Xu H. Lyu W.. (2019). Characterization of an NDM-5 carbapenemase-producing *Escherichia coli* ST156 isolate from a poultry farm in Zhejiang, China. BMC Microbiol. 19:82. doi: 10.1186/s12866-019-1454-2, PMID: 31023222 PMC6482550

[ref40] Tkadlec J. Kalova A. Brajerova M. Gelbicova T. Karpiskova R. Smelikova E.. (2021). The intestinal carriage of plasmid-mediated colistin-resistant Enterobacteriaceae in tertiary care settings. Antibiotics 10:258. doi: 10.3390/antibiotics10030258, PMID: 33806455 PMC8002115

[ref41] Uddin M. B. Alam M. N. Hasan M. Hossain S. B. Debnath M. Begum R.. (2022). Molecular detection of Colistin resistance mcr-1 gene in multidrug-resistant *Escherichia coli* isolated from chicken. Antibiotics 11:97. doi: 10.3390/antibiotics11010097, PMID: 35052973 PMC8772701

[ref42] Uelze L. Grützke J. Borowiak M. Hammerl J. A. Juraschek K. Deneke C.. (2020). Typing methods based on whole genome sequencing data. One Health Outlook 2, 1–19. doi: 10.1186/s42522-020-0010-1, PMID: 33829127 PMC7993478

[ref43] Ulrich J. U. (2023). Advanced methods for real-time metagenomic analysis of Nanopore sequencing data (doctoral dissertation).

[ref44] Umair M. Hassan B. Farzana R. Ali Q. Sands K. Mathias J.. (2023). International manufacturing and trade in colistin, its implications in colistin resistance and one health global policies: a microbiological, economic, and anthropological study. The Lancet Microbe 4, e264–e276. doi: 10.1016/S2666-5247(22)00387-1, PMID: 36931291

[ref45] Usui M. Nozawa Y. Fukuda A. Sato T. Yamada M. Makita K.. (2021). Decreased colistin resistance and mcr-1 prevalence in pig-derived *Escherichia coli* in Japan after banning colistin as a feed additive. J. Glob. Antimicrob. Resist. 24, 383–386. doi: 10.1016/j.jgar.2021.01.016, PMID: 33545419

[ref46] van Den Bunt G. Fluit A. C. Bootsma M. C. van Duijkeren E. Scharringa J. van Pelt W.. (2020). Dynamics of intestinal carriage of extended-Spectrum Beta-lactamase–producing Enterobacteriaceae in the Dutch general population, 2014–2016. Clin. Infect. Dis. 71, 1847–1855. doi: 10.1093/cid/ciz1091, PMID: 31688916

[ref47] Vu B. le T. Nguyen M. Nguyen T. Nguyen D. le D.. (2022). Characterization of genetic elements carrying mcr-1 gene in *Escherichia coli* from the community and hospital settings in Vietnam. Microbiology spectrum 10, e01356–e01321. doi: 10.1128/spectrum.01356-21, PMID: 35138158 PMC8826730

[ref48] Wang Y. Xu C. Zhang R. Chen Y. Shen Y. Hu F.. (2020). Changes in colistin resistance and mcr-1 abundance in *Escherichia coli* of animal and human origins following the ban of colistin-positive additives in China: an epidemiological comparative study. Lancet Infect. Dis. 20, 1161–1171. doi: 10.1016/S1473-3099(20)30149-3, PMID: 32505232

[ref49] Wise M. G. Estabrook M. A. Sahm D. F. Stone G. G. Kazmierczak K. M. (2018). Prevalence of mcr-type genes among colistin-resistant Enterobacteriaceae collected in 2014-2016 as part of the INFORM global surveillance program. PLoS One 13:e0195281. doi: 10.1371/journal.pone.0195281, PMID: 29608599 PMC5880376

[ref50] Xie J. Liang B. Xu X. Yang L. Li H. Li P.. (2022). Identification of mcr-1-positive multidrug-resistant *Escherichia coli* isolates from clinical samples in Shanghai, China. J. Global Antimicrobial Resistance 29, 88–96. doi: 10.1016/j.jgar.2022.02.008, PMID: 35182776

[ref51] Zahra R. Javeed S. Malala B. Babenko D. Toleman M. A. (2018). Analysis of *Escherichia coli* STs and resistance mechanisms in sewage from Islamabad, Pakistan indicates a difference in *E. coli* carriage types between South Asia and Europe. J. Antimicrob. Chemother. 73, 1781–1785. doi: 10.1093/jac/dky109, PMID: 29648612

[ref52] Zhang H. Yu F. Lu X. Li Y. Peng D. Wang Z.. (2022). Rapid detection of MCR-mediated colistin resistance in *Escherichia coli*. Microbiol Spectrum 10, e00920–e00922. doi: 10.1128/spectrum.00920-22, PMID: 35616398 PMC9241874

