# Supplementary material for: Dissemination of clinical Escherichia coli harboring the mcr-1 gene in Pakistan
Source: Front Microbiol. 2025 Jan 7;15:1502528. doi: 10.3389/fmicb.2024.1502528 (PMC11747048; doi:10.3389/fmicb.2024.1502528)
Supplement: Supplementary file 1 [file Table_1.docx]

**Table 1: Phenotypic characteristics of colistin resistant clinical *E. coli* isolates**

| **Isolate**  **ID** | **Isolation**  **date** | **Source** | **Gender** | **Age**  **(Years)** | **MIC**  **Colistin** |
| --- | --- | --- | --- | --- | --- |
| PK-5073 | 16/12/2019 | Urine | F | 44 | 8μg/ml |
| PK-5088 | 23/12/2019 | Pus | F | 47 | 8μg/ml |
| PK-5090 | 27/12/2019 | Urine | M | 19 | 8μg/ml |
| PK-5121 | 6/2/2020 | Urine | F | 52 | 32μg/ml |
| PK-5139 | 21/2/2020 | Urine | M | 46 | 16μg/ml |
| PK-5163 | 14/8/2020 | Urine | M | 69 | 16μg/ml |
| PK-5185 | 25/9/2020 | Pus | M | 25 | 32μg/ml |
| PK-5199 | 27/10/2020 | Urine | F | 37 | 16μg/ml |
| PK-5205 | 16/11/2020 | Urine | F | 62 | 32μg/ml |
| PK-5235 | 15/12/2020 | Pus | F | 43 | 16μg/ml |
