# Supplementary material for: Dissemination of clinical Escherichia coli harboring the mcr-1 gene in Pakistan
Source: Front Microbiol. 2025 Jan 7;15:1502528. doi: 10.3389/fmicb.2024.1502528 (PMC11747048; doi:10.3389/fmicb.2024.1502528)
Supplement: Supplementary file 2 [file Table_2.docx]

**Table 2: Antimicrobial Susceptibility Testing**

| **Isolate IDs** | **Cefuroxime (CXM)** | **Cefotaxime (CTX)** | **Ceftazidime (CAZ)** | **Ciprofloxacin (CIP)** | **Mecillinam (MEL)** | **Ampicillin (AMP)** | **Amoxiclav (AMC)** | **Meropenem (MEM)** | **Imipenem (IPM)** | **Amikacin (AK)** | **Tetracycline (TE)** | **Trimethoprim (TM)** |
| --- | --- | --- | --- | --- | --- | --- | --- | --- | --- | --- | --- | --- |
| PK-5073 | R | S | S | R | S | R | S | S | S | S | R | R |
| PK-5088 | R | S | S | R | R | R | S | S | S | R | R | R |
| PK-5090 | R | R | S | R | S | R | S | S | S | S | R | R |
| PK-5121 | S | S | S | R | S | R | S | S | S | S | R | R |
| PK-5139 | R | S | S | R | R | R | S | S | S | S | R | R |
| PK-5163 | S | S | S | R | S | R | S | S | S | S | R | R |
| PK-5185 | S | S | S | R | R | R | S | S | S | S | R | S |
| PK-5199 | R | S | S | R | R | R | R | S | S | S | R | R |
| PK-5205 | S | S | S | R | S | R | S | S | S | S | R | R |
| PK-5235 | R | R | R | R | S | R | R | S | S | R | R | S |
